# Container-Number Detection and Recognition in Land-Port Yard Scenes Based on an Improved YOLO11 Detector and a Lightweight CRNN Recognizer

**DOI:** 10.3390/s26196014

**Published:** 2026-09-23

**Authors:** Yuan Wu, Yongman Zhao, Yating Li, Huibing Wang, Jingqi Gong, Li Han

**Affiliations:** 1 College of Mechanical and Electrical Engineering, Shihezi University, Shihezi 832003, China; wuyuan1@xjshzu.com (Y.W.); liyating98@xjshzu.com (Y.L.); wanghuibing@xjshzu.com (H.W.); gongjingqi@xjshzu.com (J.G.); 2 Xinjiang Huiding Logistics Co., Ltd., Shihezi 832000, China; hanli@xj-hdwl.com

**Keywords:** container number recognition, neural network, object detection, deep learning

## Abstract

Automatic container-number recognition in land-port yards is challenged by clutter, illumination changes, scale variation, surface wear, and character distortion. This paper proposes a two-stage detection–cropping–recognition framework comprising an improved YOLO11 detector and a lightweight convolutional recurrent neural network (CRNN). The detector integrates C3k2 with channel-split multiscale aggregation (C3k2-CSMA), a hierarchical efficient feature fusion pyramid network (HEFFPN), selective boundary aggregation (SBA), and a shared enhanced detection head (SED-Head) with detail-enhanced convolution (DEConv) to improve small-target representation, feature fusion, and boundary localization. The recognizer combines MobileNetV3 with bidirectional long short-term memory (Bi-LSTM) and is trained using connectionist temporal classification (CTC) loss and L2 regularization. The detector achieved 97.3% mean average precision at an intersection-over-union threshold of 0.5 (mAP@0.5), 97.1% recall, 55 frames per second (FPS), and a model size of 12.2 MB. The recognizer achieved 97.6% exact-match accuracy, a model size of 6.2 MB, and an inference time of 8.9 ms/crop. The complete system achieved 96.8% end-to-end exact-match accuracy, a total model size of 18.4 MB, and a total inference time of 27.1 ms/image, demonstrating a practical accuracy–efficiency balance for the evaluated complex land-port scenes.

## 1. Introduction

Container numbers provide standardized identification information for freight containers [1]. In land-port operations, automatic container-number detection and recognition can support entry and exit records, yard handling, scheduling, and traceability by providing structured identification results [2,3,4]. Manual reading and transcription are time-consuming and susceptible to recording errors, particularly when container traffic is intensive or images must be processed continuously.

Images acquired in land-port yard scenes may be affected by cluttered backgrounds, illumination variation, scale variation, surface wear, partial occlusion, and character distortion [2,4,5]. Corrugated container panels, door-lock contours, and non-target markings can produce edges or character-like patterns that are easily confused with container-number regions. Illumination variation, surface wear, partial occlusion, and character distortion can weaken character boundaries and stroke responses, while scale variation may cause features of small container-number regions to be lost during downsampling. These factors may lead to localization offsets or missed detections, with the resulting errors propagating to subsequent sequence recognition.

Traditional image-processing methods rely on manually designed features, rules, thresholds, and often explicit character segmentation, which makes them sensitive to illumination changes and background variation [6]. General-purpose object detectors may not simultaneously preserve spatial details for small targets and suppress interference from complex container structures [3,4,7]. Modern scene-text recognizers use recurrent, attention-based, Transformer-based, or language-modeling components [8,9,10,11,12,13]; these alternatives involve different accuracy–efficiency trade-offs when lightweight deployment is required. In a two-stage pipeline, incomplete or inaccurate localization can further affect recognition because the recognizer operates on the cropped detection result [3,4]. Therefore, container-number detection and recognition require coordinated consideration of multiscale localization, boundary-relevant feature selection, sequence modeling, and computational efficiency. The relevant developments in container-number recognition, YOLO-based detection, multiscale fusion, and lightweight architectures are reviewed in Section 2.

Accordingly, this study adopts a two-stage detection–cropping–recognition framework comprising an improved YOLO11 detector and a lightweight CRNN recognizer. In the detection stage, C3k2-CSMA is designed to enrich multiscale feature representation, while the P2 detection layer and HEFFPN are designed to preserve spatial and multiscale information for small container-number regions. SBA is embedded in the HEFFPN fusion stage and aims to refine boundary-relevant responses while suppressing interference from container structures and non-target markings. SED-Head facilitates shared detection processing across P2, P3, and P4, and DEConv is incorporated into its shared convolutional stem to enhance local detail representation during training. In the recognition stage, the MobileNetV3–Bi-LSTM–CTC recognizer is designed to balance lightweight feature extraction and sequence modeling.

Within this scope, the main contributions are summarized as follows:

(1) A two-stage detection–cropping–recognition framework is proposed for container-number detection and recognition in complex land-port yard scenes. The framework separates container-number region localization from full-sequence recognition and facilitates evaluation of the detection branch, recognition branch, and complete system within a consistent task pipeline.

(2) An improved YOLO11 detector is developed by combining C3k2-CSMA, a P2–P3–P4 HEFFPN with SBA, and SED-Head with DEConv. These components are designed to facilitate multiscale feature representation, help preserve spatial information for small container-number regions, refine boundary-relevant responses, and support shared detection processing.

(3) A lightweight MobileNetV3–Bi-LSTM–CTC recognizer is constructed for full-sequence container-number recognition. The detector, recognizer, and complete two-stage system were evaluated under the considered land-port yard conditions.

## 2. Related Work

### 2.1. Container-Number and Scene-Text Recognition

Early container-number recognition systems typically decomposed the task into image enhancement, region localization, character segmentation, and character classification. Edge operators, thresholding, mathematical morphology, connected components, projections, and template matching were practical when viewpoints and image quality were controlled [2,6]. Their dependence on handcrafted features and fixed thresholds, however, makes segmentation unstable when container surfaces are worn, characters are partly occluded, illumination is uneven, or door hardware and corrugations create character-like structures. Errors introduced during region extraction or character segmentation can propagate through subsequent recognition stages.

Deep learning shifted container-code processing toward learned localization and recognition. Container-specific studies have used adaptive localization, two-stage detection–recognition pipelines, confidence-guided recognition, and DETR-based character recognition to reduce reliance on manually specified rules [3,4,5,7]. These approaches improve the ability to learn visual representations from data, but they retain an upstream–downstream dependency between the detector and recognizer: an offset, truncated crop, or missed container-number region still limits the information available to the sequence model. This issue is particularly relevant to exact sequence output because one localization error can affect several adjacent characters.

Scene-text recognition provides complementary strategies for decoding cropped text. CRNN combines convolutional features with recurrent sequence modeling, while CTC permits training without explicit character-level alignment [9,14]. Attention-based rectification and decoding can accommodate irregular text geometry [10], whereas SVTR, PARSeq, and ABINet use visual Transformers, autoregressive ordering, or iterative language modeling to capture broader contextual relationships [11,12,13]. Such mechanisms are valuable for distorted or ambiguous text, but rectification, attention, and language-modeling components can increase architectural and implementation complexity relative to compact convolutional–recurrent recognizers. For ISO 6346 container codes, the restricted alphabet and prescribed sequence structure [1] make it useful to combine lightweight visual feature extraction, bidirectional sequence modeling, and alignment-free CTC decoding, while retaining awareness that detection errors can propagate to recognition.

### 2.2. YOLO-Based Detection in Challenging Scenes

YOLO formulates object detection as direct prediction of bounding boxes and class probabilities from an image in a unified network [15]. Later implementations, including YOLOv5, YOLOv8, and YOLO11, retain the one-stage detection principle while revising the backbone, feature-fusion path, prediction head, and training strategy [16,17,18]. Their accuracy–efficiency balance makes them useful baselines for industrial vision, but a general detector must still be adapted to the geometry and imaging conditions of the target domain. Container-number regions are elongated and may occupy only a small portion of a yard image; repeated downsampling can weaken character boundaries, local spacing, and other spatial cues needed for reliable localization.

Feature pyramids and path aggregation address scale variation by combining shallow spatial detail with deeper semantic information [19,20]. For small targets, retaining a high-resolution shallow feature level can therefore complement the conventional coarser detection scales. Recent remote-sensing work combines feature weaving with redundancy suppression to coordinate information across scales while suppressing redundant feature responses [21]. Although remote-sensing objects differ from container-number strips, this study is relevant to the general problem of preserving multiscale information for small targets and controlling feature redundancy. It does not, by itself, establish performance for land-port yard scenes.

Illumination variation presents a related but distinct challenge. AMIEOD jointly optimizes multi-expert image enhancement and object detection, using detection-oriented objectives to select enhancement strategies for low-illumination inputs [22]. This work shows that enhancement and detection can be coupled when low-light degradation is the primary concern; it does not imply that an additional enhancement module is necessary for every container-number pipeline. In land-port images, low illumination, strong reflection, door-lock contours, corrugated panels, and non-target markings may occur together. General-purpose YOLO detectors are not necessarily optimized for the weak and elongated boundaries of container-number regions [3,4,7]. These observations motivate the use of multiscale detection and shallow high-resolution features, together with feature refinement targeted at boundary-related interference.

### 2.3. Multiscale Feature Fusion and Lightweight Architectures

Multiscale feature fusion is central to detecting objects whose apparent sizes vary across images. FPN uses lateral connections and a top-down pathway to combine semantic and spatial information, while PANet adds a bottom-up aggregation path to shorten information flow between low and high feature levels [19,20]. These designs provide a general basis for small-target detection, but direct aggregation can also transmit background textures and redundant responses. Attention-based selection can reweight channels and spatial locations [23], and boundary-aware networks demonstrate that shape-related cues can be separated and refined rather than treated as ordinary semantic features [24]. For container-number detection, this distinction is important because panel seams and door-lock structures can create strong edges that do not correspond to the target boundary.

Efficiency-oriented architectures reduce repeated computation through compact feature blocks, shared processing, and parameter reuse. Lightweight detectors based on feature fusion illustrate that the neck and head must be considered together when balancing localization quality and computational cost [25]. Shared detection heads can reuse common transformations across scales, although scale-specific classification and box regression must still be preserved where required. Structural reparameterization offers another route: RepVGG uses a multi-branch training structure that can be converted to a simpler convolutional form for inference [26]. Detail-enhanced convolution similarly emphasizes local gradients and texture information in its original image-restoration context [27]. These studies support the general rationale for training-time feature enrichment and inference-time simplification, but their effects must be verified for each detection task rather than assumed from architecture alone.

Lightweight sequence recognition requires the same caution. MobileNetV3 was developed to provide efficient visual feature extraction under resource constraints [28], whereas bidirectional recurrent networks model context from both sequence directions [29]. When combined with CRNN and CTC principles [9,14], these elements offer a compact route from a cropped container-number image to a complete character sequence without explicit character segmentation. The resulting design objective is not to maximize recognition-model complexity, but to retain sufficient local detail and sequence context while controlling storage and inference burden.

Motivated by these observations, existing work provides complementary foundations in one-stage detection, multiscale fusion, boundary-aware refinement, structural reparameterization, and scene-text recognition. Complex land-port yard scenes nevertheless require small-target localization, suppression of boundary-related interference, multiscale feature fusion, and lightweight sequence recognition to be considered within one pipeline. Accordingly, this study investigates a two-stage detection–cropping–recognition framework composed of an improved YOLO11 detector and a lightweight CRNN recognizer.

## 3. Materials and Methods

### 3.1. Overall Framework

A two-stage detection–cropping–recognition framework is adopted, as illustrated in Figure 1. The original yard image is first fed into the improved YOLO11 detection model [16] to obtain the bounding box of the complete 11-character container-number strip region. The predicted region is then cropped and resized to the input dimensions required by the recognition model. Finally, the lightweight CRNN [9] performs feature extraction, sequence modeling, and CTC decoding [14] on the cropped strip image to output the complete 11-character container number.

The detection branch localizes the container-number region and thereby reduces the amount of background content passed to the recognition branch. The restricted character set and fixed-length format provide task-specific context for interpreting full-sequence outputs. Compared with direct optical character recognition on the entire image, the two-stage architecture reduces the influence of cluttered backgrounds on the recognizer and enables detection errors, recognition errors, and end-to-end errors to be analyzed separately.

The detection and recognition subnetworks were optimized independently rather than jointly in an end-to-end manner. The detector was trained using yard images and bounding-box annotations, whereas the recognizer was trained using cropped container-number strips and their sequence labels. During inference, the bounding box predicted by the detector determines the region cropped and resized for the recognizer. The recognition loss is therefore not back-propagated through the cropping operation to update the detector. This design permits separate evaluation of the two stages while retaining the practical upstream–downstream dependency of the complete system.

The components of the proposed framework perform complementary functions rather than providing one-to-one guarantees for individual scene conditions. In the backbone, one C3k2-CSMA module replaces the first C3k2 layer immediately after the P2/4 downsampling convolution. Its hierarchical 3 × 3, 5 × 5, and 7 × 7 grouped convolutions are designed to represent complementary patterns associated with character strokes, character groups, and the complete container-number strip. HEFFPN retains the C5/P5 backbone feature as a semantic input, performs top-down fusion from P5 to P2 and bottom-up fusion from P2 to P4, and supplies P2, P3, and P4 features to SED-Head. The high-resolution P2 output helps preserve spatial information for small targets, whereas bidirectional fusion facilitates feature exchange under scale variation. SBA remains confined to the HEFFPN fusion node that generates P2, where it derives a boundary cue internally from the aligned low-level feature. DEConv remains within SED-Head, whose shared transformations operate across the P2, P3, and P4 detection scales. After cropping, MobileNetV3 extracts lightweight visual features, Bi-LSTM models contextual relationships in both sequence directions, and CTC produces the output without requiring character-level alignment. No dedicated illumination-enhancement branch is introduced. These mechanisms are intended to mitigate, rather than eliminate, the effects of clutter, weak boundaries, scale variation, surface wear, partial occlusion, and character distortion.

### 3.2. Improved YOLO11 Detection Model

YOLO11n [16] is adopted as the detection baseline and structurally adapted to the elongated geometry, scale variation, weak boundaries, and structural interference associated with container-number regions. A single C3k2-CSMA module replaces the first C3k2 layer after the P2/4 downsampling convolution and produces the C2 feature used by the neck. HEFFPN receives C2, C3, C4, and the retained C5/P5 backbone feature, aligns these features to 64 channels, and constructs a P5-to-P2 top-down path followed by a P2-to-P4 bottom-up path. The P5 backbone feature provides deep semantic information for fusion, but the proposed detector does not use a P5 detection head. SBA is placed only at the HEFFPN node that generates T2, and the final T2, B3, and B4 features form the P2, P3, and P4 inputs to SED-Head. DEConv remains in the shared convolutional stem of SED-Head. These changes are intended to support small-target localization and multiscale feature use while keeping every custom component at a defined position in the detection graph.

#### 3.2.1. C3k2-CSMA Backbone Module

Container-number regions generally have an elongated geometry and may occupy only a small proportion of a yard image. Repeated downsampling can weaken character strokes, inter-character spacing, and strip boundaries, particularly for distant targets. The first C3k2 layer immediately following the P2/4 downsampling convolution is therefore replaced by one C3k2-CSMA module. This single insertion is designed to enrich local-detail and multiscale structural representations at the 160 × 160 backbone stage without applying the replacement to later C3k2 layers.

Under nano-scale width and depth scaling, the module receives an input tensor of *N* × 32 × 160 × 160 and produces an output tensor of *N* × 64 × 160 × 160, with one module repetition. The fixed branch-width ratio *ρ* = 0.25 gives a minimum branch width C_min_ = 16 channels. As shown in Figure 2, a 3 × 3 grouped convolution first maps the 32-channel input to 64 channels. An equal channel split retains one 32-channel subset and sends the other 32-channel subset to a 5 × 5 grouped convolution. The resulting 32 channels are split equally again: one 16-channel subset is retained, and the other is processed by a 7 × 7 grouped convolution from 16 to 16 channels. The retained 32-channel subset, retained 16-channel subset, and transformed 16-channel subset are concatenated to form 64 channels. The 3 × 3, 5 × 5, and 7 × 7 convolutions use groups = 2, stride = 1, and paddings of 1, 2, and 3, respectively; each is followed by BatchNorm2d and SiLU. A bias-free 1 × 1 convolution followed by BatchNorm2d(64) fuses the concatenated feature. In parallel, a bias-free 1 × 1 projection followed by BatchNorm2d(64) maps the 32-channel input to 64 channels. The main and shortcut paths are added, and SiLU produces the module output.

The successive equal splits restrict larger-kernel processing to progressively narrower feature subsets rather than applying every spatial convolution to the complete input tensor. The retained subsets provide direct routes to the concatenation, while the projected shortcut provides a separate information and gradient path across the module. The concatenation, 1 × 1 fusion, residual addition, and final activation close the 32 → 64 → 32/16/16 → 64 channel flow. The value groups = 2 is a fixed implementation setting, whereas the branch-width ratio *ρ* = 0.25 was selected from the sensitivity experiment reported in Section 4.6. The module’s net computational effect is interpreted together with the complete-detector complexity measurements.

The hierarchical 3 × 3, 5 × 5, and 7 × 7 operations are intended to provide complementary receptive fields for patterns associated with individual strokes, character groups, and the complete strip. Their use at the C2 stage also helps preserve information before deeper downsampling. These roles describe the design rationale; the resulting effects on detection accuracy and model complexity are assessed at the detector level in Section 4.

#### 3.2.2. HEFFPN and Detection Layer Reconfiguration

The baseline YOLO11n detector produces predictions at the P3, P4, and P5 scales. The proposed detector retains the P5 backbone stage and its deep semantic feature but removes the P5 detection head; the P5 feature is used only as the semantic input to the HEFFPN top-down path. For a 640 × 640 detector input, HEFFPN receives C2: *N* × 64 × 160 × 160, C3: *N* × 128 × 80 × 80, C4: *N* × 128 × 40 × 40, and C5: *N* × 256 × 20 × 20. C2 is produced by the single C3k2-CSMA module described in Section 3.2.1, whereas C5 is the feature obtained after deep semantic processing in the retained P5 backbone stage.

Each input *C_i_* is mapped to 64 channels by a bias-free 1 × 1 Conv–BN–SiLU alignment transform *A_i_*. The aligned features are L2: *N* × 64 × 160 × 160, L3: *N* × 64 × 80 × 80, L4: *N* × 64 × 40 × 40, and L5: *N* × 64 × 20 × 20.

$$L_{i}=A_{i}(C_{i}),\;i\in \{2,3,4,5\}$$

The top-down path proceeds in the order P5 → P4 → P3 → P2. Up2 denotes twofold nearest-neighbor upsampling. At the P4 and P3 fusion nodes, the upsampled higher-level feature is concatenated with the corresponding lateral feature, giving 128 channels. Bias-free 1 × 1 Conv–BN–SiLU transforms Φ4 and Φ3 compress these concatenated features to 64 channels. At the high-resolution P2 node, SBA receives Up2(T3) as the aligned high-level input and L2 as the aligned low-level input and produces T2. This is the only SBA insertion in HEFFPN.

$$T_{4}=\Phi_{4}(\mathrm{Concat}[L_{4},{\mathrm{Up}}_{2}(L_{5})]),\;T_{3}=\Phi_{3}(\mathrm{Concat}[L_{3},{\mathrm{Up}}_{2}(T_{4})]),\;T_{2}=SBA({\mathrm{Up}}_{2}(T_{3}),L_{2})$$

The bottom-up path proceeds in the order P2 → P3 → P4. Each Down2 operation is a bias-free 3 × 3 Conv–BN–SiLU with stride = 2 and padding = 1. The downsampled feature is concatenated with the top-down feature at the same resolution, and bias-free 1 × 1 Conv–BN–SiLU transforms Ψ3 and Ψ4 compress the resulting 128 channels to 64. T2 enters both the P2 detection path and the bottom-up path. The final HEFFPN outputs supplied to the shared SED-Head are T2: N × 64 × 160 × 160, B3: N × 64 × 80 × 80, and B4: N × 64 × 40 × 40, corresponding to the P2, P3, and P4 scales, respectively.

$$B_{3}=\Psi_{3}(Concat[T_{3},{Down}_{2}(T_{2})]),\;B_{4}=\Psi_{4}(Concat[T_{4},{Down}_{2}(B_{3})])$$

$$Y=SEDHead(T_{2},B_{3},B_{4})$$

Figure 3 summarizes this feature graph. The high-resolution T2 output helps preserve spatial detail for small container-number regions, while the retained C5/P5 feature and the bidirectional P2–P4 fusion paths provide broader semantic and scale context. The final HEFFPN outputs T2, B3, and B4 are supplied to the shared SED-Head at the P2, P3, and P4 scales, respectively. No P5 detection head is used. SBA refines only the P2 fusion node and does not act as an independent prediction layer.

Introducing the P2 prediction scale increases the spatial computation of the high-resolution fusion and detection paths. Removing the P5 detection head, compressing each fusion result to 64 channels, and applying the subsequent shared detection head are intended to limit repeated processing. The P2–P3–P4 configuration should therefore be interpreted as an accuracy–efficiency trade-off rather than a cost-free modification. Its net computational effect cannot be determined from the topology alone and is considered at the complete-detector level.

#### 3.2.3. SBA Module

Cross-scale fusion at the high-resolution P2 node can transmit both target-related information and high-frequency responses produced by container corrugations, door-lock contours, and non-target markings. SBA is therefore placed only at the HEFFPN fusion node that generates P2. Before SBA, the upsampled P3 high-level feature and the P2 low-level feature are aligned by the HEFFPN fusion node to 64 channels. The aligned tensors are denoted by ${\hat{F}}_{h},{\hat{F}}_{l}\in R^{N\times 64\times H\times W}$; for a 640 × 640 detector input, *H* = *W* = 160. The feature-alignment convolutions belong to HEFFPN rather than to the SBA body.

Within SBA, the boundary cue is generated from the aligned low-level feature rather than supplied as a separate input. Fixed depthwise 3 × 3 Sobel kernels are applied independently to the 64 channels with groups = 64:

$$E_{x}={DWConv}_{S_{x}}({\hat{F}}_{l}),E_{y}={DWConv}_{S_{y}}({\hat{F}}_{l})$$

$$\bar{E}={Mean}_{c}(\sqrt{{E_{x}}^{2}+{E_{y}}^{2}+\varepsilon })$$

$$B=Sigmoid(\frac{\bar{E}-\mu_{HW}(\bar{E})}{\sqrt{{var}_{HW}(\bar{E})+\varepsilon }})$$

Here, *S_x_* and *S_y_* are fixed Sobel kernels and are not updated during training. Mean*_c_*(·) averages the channel dimension, whereas *μ_HW_*(·) and var*_HW_*(·) are calculated separately for each sample over the *H* × *W* spatial dimensions. The numerical constant is *ε* = 1 × 10^−6^, and the resulting boundary cue satisfies $B\;\in R^{N\times 1\times H\times W}.$

The single-channel cue is converted into the boundary-guidance feature by a 1 × 1 convolution from 1 to 64 channels without bias, followed by BatchNorm2d(64) and SiLU:

$$G\;=SiLU(BN({{Conv}_{1\times 1}}^{1\to 64}(B))),\;G\in R^{N\times 64\times H\times W}$$

For feature selection, the aligned high- and low-level tensors are first added to obtain Z. The channel-wise concatenation [Z,G] is then processed by a bias-free 1 × 1 convolution from 128 to 64 channels, batch normalization, and a sigmoid function to produce the gate M:

$$Z\;={\hat{F}}_{h}+{\hat{F}}_{l},\;M=Sigmoid(BN({{Conv}_{1\times 1}}^{128\to 64}([Z,G]))),\;M\in R^{N\times 64\times H\times W}$$

The gate balances the two aligned inputs, after which G modulates the selected representation without being interpreted as a boundary probability map or a standalone attention output:

$$F_{sel}=M⊙{\hat{F}}_{l}+(1-M)⊙{\hat{F}}_{h},F_{g}=F_{sel}⊙(1+G)$$

Selective boundary aggregation applies DSConv to the modulated feature. DSConv comprises a bias-free 3 × 3 depthwise convolution from 64 to 64 channels with groups = 64 and padding = 1, followed by BatchNorm2d(64) and SiLU, and then a bias-free 1 × 1 pointwise convolution from 64 to 64 channels followed by BatchNorm2d(64) and SiLU. Its residual output is

$$R=DSConv(F_{g}),F_{SBA}=F_{sel}+R$$

The resulting *F*_SBA_ is supplied to the subsequent P2 detection path and the bottom-up HEFFPN fusion path. The cue is computed directly from the aligned low-level tensor and is optimized solely through the detector loss; no additional boundary annotations or loss terms are introduced. Although the Sobel kernel values remain fixed, the aligned low-level tensor is not detached. Consequently, the detector loss can propagate gradients through the Sobel response, spatial normalization, guidance, gating, DSConv, and residual paths to the low-level input feature. The direct high- and low-level routes and the residual addition provide complementary information and gradient paths. SBA adds processing only at this P2 fusion node and does not independently produce classification scores or bounding-box regression outputs. It is designed to refine target-related boundary and stroke responses rather than eliminate structural interference, and its detector-level effect is evaluated in Section 4.

#### 3.2.4. SED-Head and DEConv

The three HEFFPN outputs enter SED-Head as T2: *N* × 64 × 160 × 160 at P2, B3: *N* × 64 × 80 × 80 at P3, and B4: *N* × 64 × 40 × 40 at P4. The same shared_stem instance is invoked for the three inputs in turn. Consequently, the DEConv parameters and the parameters of the remaining convolution and batch-normalization layers are shared across P2, P3, and P4, while the same parameter-free SiLU operations are applied at all three scales. Each scale retains its own spatial resolution throughout this shared transformation.

As shown in Figure 4b, shared_stem applies the following fixed sequence: (1) a bias-free 1 × 1 Conv–BN–SiLU layer from 64 to 64 channels with stride = 1, padding = 0, and groups = 1; (2) a 3 × 3 DEConv from 64 to 64 channels with stride = 1, padding = 1, groups = 1, and bias enabled; (3) a bias-free 3 × 3 depthwise Conv–BN–SiLU layer from 64 to 64 channels with stride = 1, padding = 1, and groups = 64; and (4) a bias-free 1 × 1 Conv–BN–SiLU layer from 64 to 64 channels with stride = 1, padding = 0, and groups = 1. DEConv contains no internal batch-normalization or activation layer, and its output is supplied directly to the subsequent 3 × 3 depthwise Conv–BN–SiLU layer.

Let *P_i_* denote T2, B3, or B4 for i ∈ {2, 3, 4}, and let *S_i_* be the corresponding shared-stem output. After shared processing, every scale uses its own classification and regression output layers; these output-layer parameters are not shared across scales. The classification layer is Conv2d(64 → 1, kernel = 1, stride = 1, padding = 0, bias = True), and the regression layer is Conv2d(64 → 64, kernel = 1, stride = 1, padding = 0, bias = True). No additional learnable regression scaling factor is used.

$$S_{i}=S(P_{i}),\;i\in \{2,3,4\}$$

$$C_{i}={ClsConv}_{i}(S_{i}),\;shape(C_{i})=N\times 1\times H_{i}\times W_{i}$$

$$R_{i}={RegConv}_{i}(S_{i}),\;shape(R_{i})=N\times 64\times H_{i}\times W_{i}$$

$$O_{i}=Concat[R_{i},C_{i}],\;shape(O_{i})=N\times 65\times H_{i}\times W_{i},\;i\in \{2,3,4\}$$

The detector uses one class (nc = 1), an anchor-free prediction formulation, reg_max = 16, and Distribution Focal Loss (DFL), with strides of 4, 8, and 16 for P2, P3, and P4, respectively. Accordingly, the raw prediction tensors are *N* × 65 × 160 × 160, *N* × 65 × 80 × 80, and *N* × 65 × 40 × 40. At each location, 64 channels contain the 4 × reg_max regression-distribution logits and one channel contains the classification logit. There is no separate objectness output. These tensors are raw predictions rather than final boxes; DFL projection and grid-based decoding subsequently convert the regression distributions into bounding boxes.

DEConv is the second operation in shared_stem and follows the detail-enhanced convolution principle in [27]. It contains five independently trained operators: vanilla convolution (VC), central-difference convolution (CDC), angular-difference convolution (ADC), horizontal-difference convolution (HDC), and vertical-difference convolution (VDC). VC is the intensity-preserving operator; CDC and ADC are local-difference detail operators; and HDC and VDC are directional-gradient operators. These three functional categories comprise five distinct parameter sets. VC, CDC, and ADC each use a trainable base weight of size 64 × 64 × 3 × 3. HDC and VDC each use trainable directional parameters of size 64 × 64 × 3, which are embedded into a 3 × 3 equivalent kernel by fixed placement rules. Every operator has an independent trainable bias. No operator contains a separate batch-normalization or activation layer, and none uses fixed Sobel, Prewitt, or Scharr coefficient values.

For a fixed output–input channel pair, let q ∈ {0, …, 8} index the 3 × 3 kernel positions in row-major order, and let W_o denote the trainable base weights of operator o. VC retains its base kernel. CDC retains all noncentral coefficients and replaces the center coefficient by the base center minus the sum of all nine base coefficients:

$$K_{VC}[q]=W_{VC}[q],\;q=0,\ldots ,8$$

$$S_{CDC}=W_{CDC}[0]+W_{CDC}[1]+\ldots +W_{CDC}[8]$$

$$K_{CDC}[q]=W_{CDC}[q]\;(q\neq 4),\;K_{CDC}[4]=W_{CDC}[4]-S_{CDC}$$

For ADC, the fixed permutation is *π* = (3, 0, 1, 6, 4, 2, 7, 8, 5), where *π*(q) gives the paired source position for output position q. The difference coefficient is formed with *θ* = 1.0; *θ* is fixed rather than learnable:

$$K_{ADC}[q]=W_{ADC}[q]-\theta \;W_{ADC}[\pi (q)],\;\theta =1.0$$

For each output–input channel pair, let h = (h0, h1, h2) and v = (v0, v1, v2) denote the three trainable directional coefficients of HDC and VDC, respectively. HDC places the positive coefficients at flattened positions (0, 3, 6) and their negatives at (2, 5, 8), whereas VDC uses positive positions (0, 1, 2) and negative positions (6, 7, 8):

$$K_{HDC}=\left[\begin{array}{ccc} h_{0} & 0 & -h_{0}\\ h_{1} & 0 & -h_{1}\\ h_{2} & 0 & -h_{2} \end{array}\right]$$

$$K_{VDC}=\left[\begin{array}{ccc} v_{0} & v_{1} & v_{2}\\ 0 & 0 & 0\\ -v_{0} & -v_{1} & -v_{2} \end{array}\right]$$

The five equivalent kernels and their independent biases are added before the spatial convolution. With X as the DEConv input, the resulting operation is

$$K_{eq}=K_{VC}+K_{CDC}+K_{ADC}+K_{HDC}+K_{VDC}$$

$$b_{eq}=b_{VC}+b_{CDC}+b_{ADC}+b_{HDC}+b_{VDC}$$

$$Y_{DE}=Conv2d(X;\;K_{eq},\;b_{eq})$$

During training, the five trainable base parameter sets are first transformed by the above fixed difference rules into five equivalent 3 × 3 kernels. The equivalent kernels and biases are then summed, and one standard convolution generates the DEConv output; the implementation does not require five full spatial convolutions or five separately materialized output feature maps. Back-propagation through the algebraic kernel construction updates all five base parameter sets. Thus, training adds parameter storage, equivalent-kernel construction, and associated gradient paths. Before deployment, *K*_eq_ and *b*_eq_ are retained as one ordinary 3 × 3 convolution with 64 input channels, 64 output channels, stride = 1, padding = 1, groups = 1, and bias enabled. DEConv reparameterization does not include batch-normalization fusion because DEConv has no internal batch-normalization layer; any later framework-level Conv–BN fusion is a separate deployment operation. This algebraic equivalence describes the operator-level inference form and is not presented as a complete-detector validation.

### 3.3. Lightweight CRNN Recognition Model

The recognition branch operates on cropped container-number strip images. During complete-system inference, the improved YOLO11 detector localizes the container-number region, and the predicted region is cropped and directly resized to 32 × 160 pixels by bilinear interpolation. The resized strip is represented in RGB, its pixel values are scaled to [0, 1], and ImageNet mean and standard-deviation normalization is applied. The recognizer comprises a Modified MobileNetV3-Small feature extractor, a single-layer bidirectional long short-term memory (Bi-LSTM) network, a character-classification layer, and greedy connectionist temporal classification (CTC) decoding. Detection and recognition remain independently optimized, so the recognition loss is not back-propagated to the detector.

#### 3.3.1. Modified MobileNetV3-Small Feature Extraction Backbone

Conventional CRNN architectures generally use a VGG-style convolutional front end for visual feature extraction [9]. Although effective, this front end can impose a relatively large storage and computational burden in a compact recognition pipeline. In this study, the VGG16 front end is replaced by Modified MobileNetV3-Small [28] with a width multiplier of 1.0. The feature extractor was initialized using the ImageNet-1K pretrained weights of MobileNetV3-Small. All retained feature-extraction layers are fine-tuned jointly; no network stage is frozen. The original AdaptiveAvgPool2d layer and ImageNet classifier are removed, while the convolutional layers in the features component are retained.

To preserve horizontal sequence resolution, the depthwise-convolution stride in the first 5 × 5 inverted-residual block that outputs 40 channels and in the first 5 × 5 inverted-residual block that outputs 96 channels is changed from (2,2) to (2,1). The remaining channel settings, convolution kernels, squeeze-and-excitation units, and activation functions follow the original MobileNetV3-Small configuration. Consequently, an input tensor of *N* × 3 × 32 × 160 is mapped to *N* × 576 × 1 × 20, corresponding to total downsampling factors of 32 in height and 8 in width. Retaining this width-wise evidence and fine-tuning the visual features are intended to support recognition when character strokes are weakened by surface wear, low contrast, local loss, or deformation; these conditions are addressed by the complete recognizer rather than by the backbone alone. The recognition flow is summarized in Figure 5.

Let the normalized recognition input be denoted by *I* with shape *N* × 3 × 32 × 160. The Modified MobileNetV3-Small mapping Φ(·) produces a feature tensor *F* with shape *N* × 576 × 1 × 20:

(1) F=Φ(I).

Because the height of F is one and container numbers are arranged horizontally, the singleton height dimension is removed and the feature-map width is treated as the temporal dimension. For each sample n, the sample-specific feature tensor has shape 576 × 1 × 20. At width index t, the corresponding column is flattened to a 576-dimensional feature vector:

(2) $$x_{t}=\;Flatten(F_{n}[:,\;:,\;t])\;\in R^{576},\;\;\;t\;=\;1,\;2,\;\ldots ,\;T,\;\;\;T\;=\;20.$$

The ordered feature vectors form the sequence

(3) $$X=(x_{1},x_{2},...,x_{T}).$$

Across a batch, the serialized tensor has shape *N* × 20 × 576. This width-wise serialization preserves left-to-right ordering without explicit character segmentation, and a time step represents a feature-map position rather than a predefined character.

#### 3.3.2. Bi-LSTM Sequence Modeling

The serialized tensor is supplied directly to a single-layer Bi-LSTM without an additional intermediate feature-projection layer. The sequence length is T = 20, and each time step has 576 input features. The Bi-LSTM uses 112 hidden units in each direction, bidirectional processing, and dropout = 0. Bidirectional context is intended to complement locally weakened visual evidence under wear, low contrast, partial stroke loss, and character deformation. For the input sequence defined in Equation (3), the forward hidden state is expressed as

(4) $${\vec{h}}_{t}=f_{LSTM}(x_{t},{\vec{h}}_{t-1}).$$

where the indicated function denotes the forward LSTM unit. The backward hidden state is expressed as

(5) $${\overset{\leftarrow }{h}}_{t}=b_{LSTM}(x_{t},{\overset{\leftarrow }{h}}_{t+1}).$$

where the indicated function denotes the backward LSTM unit. Concatenating the 112-dimensional states from the two directions gives the contextual representation at time step *t*:

(6) $$h_{t}=[{\vec{h}}_{t};{\overset{\leftarrow }{h}}_{t}].$$

The resulting Bi-LSTM output tensor has shape *N* × 20 × 224 and is passed directly to the character-classification layer.

#### 3.3.3. CTC Loss with L2 Regularization

A Linear(224,37) layer converts the Bi-LSTM representation into logits with shape *N* × 20 × 37. The 37 classes comprise 10 digits, 26 uppercase English letters, and one CTC blank class with index 0. Before the CTC objective is evaluated, the logits are arranged as a 20 × *N* × 37 tensor. Because sequence time steps are not aligned one-to-one with character positions, CTC provides supervision without character-level alignment annotations [14]. Let *X* denote the recognition-model input sequence and *Y* the corresponding target sequence. The CTC loss is

(7) $$L_{CTC}=-lnP(Y|X).$$

where *P*(*Y*|*X*) denotes the conditional probability of the target sequence. An explicit L2 term is added to the CTC loss using *λ* = 1 × 10^−4^, while the Adam optimizer uses weight_decay = 0 [30]. The resulting training objective is

(8) $$L=L_{CTC}+\lambda ∥\theta {∥}_{2}^{2}.$$

In Equation (8), the L2 term is applied to the weight matrices in convolutional layers, squeeze-and-excitation units, the Bi-LSTM, and the Linear classifier; bias terms and BatchNorm parameters are excluded. At inference, greedy CTC decoding first merges adjacent repeated classes and then removes the blank class. Beam search, a language model, ISO-format enforcement, and check-digit correction are not used. The supervised labels contain 11 characters, but unconstrained CTC decoding does not guarantee an 11-character output. A prediction is counted as correct only when the complete decoded sequence exactly matches the 11-character ground-truth label; a different length or any character mismatch is counted as incorrect.

### 3.4. Dataset and Experimental Setup

#### 3.4.1. Dataset Description and Division

The experimental dataset consists of publicly accessible container-number image samples and additionally curated images collected from public scene-image sources. Before inclusion in the experiments, low-quality images were screened, and image formats were standardized. For detection, the complete 11-character container-number strip region was annotated as a single target bounding box. For recognition, the corresponding 11-character container number was used as the supervisory label. After annotation, bounding-box completeness, string consistency, visually similar character labels, and non-standard samples were manually reviewed to reduce the influence of label noise on experimental evaluation.

After screening, 3600 images were retained. The dataset covers representative interference factors, including distant imaging, cluttered backgrounds, illumination variation, viewpoint variation, character degradation, and partial occlusion. The dataset division is presented in Table 1. A fixed test set of 1200 images was used exclusively for final evaluation. The remaining 2400 images were divided into training and validation sets at a ratio of 8:2. The training set was used for model optimization and random augmentation, the validation set was used for model selection and hyperparameter adjustment, and the test set was excluded from training, parameter adjustment, and random augmentation.

Before dataset division, duplicate samples and obviously repeated images were screened during data curation.

The original images were divided into the training, validation, and test sets before recognition strips were generated. After the split, the ground-truth bounding boxes from each subset were used to crop its recognition strip images. Thus, no crops derived from the same original image could enter different subsets. Recognition-model training, validation, and standalone testing all use ground-truth crops from their corresponding subsets.

Recognition labels consist of four uppercase English letters followed by seven digits and contain no spaces, hyphens, or CTC blank symbols. All 3600 final labels had a length of 11, satisfied the expression [A-Z]{4}[0–9]{7}, and passed ISO 6346 check-digit verification. The check-digit test was used as a quality-control procedure; no automatic replacement was applied to the annotation files.

The scene attributes listed in Table 2 were assigned through manual inspection. Multiple adverse-condition attributes could be assigned to the same image; therefore, the sum of the attribute counts does not equal the total number of images.

Figure 6 presents representative samples involving distant small-scale targets, illumination variation, viewpoint variation, character degradation, partial occlusion, and background-structure interference. These examples illustrate several recognition difficulties represented in the dataset.

#### 3.4.2. Evaluation Metrics

Detection performance was evaluated using mAP@0.5 [31], recall, FPS, trainable Parameters (M), and model size (MB). For all evaluated deep-learning detectors, fixed-threshold recall was calculated using a confidence threshold of 0.25, a matching IoU threshold of 0.50, an NMS IoU threshold of 0.70, and max_det = 300. The mAP@0.5 calculation retained predictions from a minimum confidence of 0.001 and integrated the resulting precision-recall curve at IoU = 0.50. The traditional connected-component method did not use the learned confidence threshold; candidate regions were screened by connected-area and aspect-ratio rules and ranked by normalized template similarity, with a fixed acceptance threshold of 0.60.

The recognition metrics include exact-match accuracy, model size, and inference time per crop. Standalone recognition evaluation uses test-set strip images cropped from ground-truth bounding boxes. A prediction is counted as correct only when the entire decoded string exactly matches the corresponding 11-character label; a decoded sequence of any other length or any character mismatch is counted as incorrect [2,5]. Wilson 95% confidence intervals are reported for exact-match proportions.

End-to-end exact-match accuracy is calculated using the original test images as the evaluation units and the improved YOLO11 detector’s predicted bounding boxes as the crop source. A sample is counted as correct only when a retained detection supplies a usable crop and the complete decoded sequence exactly matches the 11-character ground-truth label. A missed detection, a prediction removed at the operating threshold, a decoded sequence with a length other than 11, or any character mismatch is counted as incorrect. Because IoU measures geometric agreement rather than character coverage, a predicted box with IoU below 0.50 may still contain all 11 characters and produce a correct sequence.

Detection-model complexity is reported as trainable Parameters (M), calculated as the sum of p.numel() over tensors with p.requires_grad = True. Model size (MB) denotes the measured size of the FP32 state_dict file in decimal megabytes and is not used as a unit for parameter count. FLOPs were measured in deployment mode with batch size 1, counting one multiply-accumulate operation as two floating-point operations. Peak allocated GPU memory was measured with torch.cuda.max_memory_allocated() after resetting the peak-memory statistics immediately before the evaluated operation.

#### 3.4.3. Experimental Environment and Training Parameters

The experimental platform and principal training settings are listed in Table 3. Detection training used Ultralytics 8.3.0 for 100 epochs with a batch size of 8, an initial learning rate of 0.01, the SGD optimizer, and a random seed of 0. Recognition training used 100 epochs, a batch size of 64, an initial learning rate of 0.001, the Adam optimizer, and a random seed of 0. The explicit L2 regularization coefficient was 1 × 10^−4^, while Adam weight_decay was set to 0.

Random data augmentation was applied only to the training sets. No random augmentation was applied to the validation or test sets.

All latency measurements were performed on an NVIDIA GeForce RTX 3060 12 GB GPU with batch size = 1 and FP32 inference. Fifty warm-up iterations preceded formal timing, and CUDA synchronization was executed immediately before and after each timed interval. Disk reading, result saving, and visualization were excluded. Across the 1200 test images, the mean detection, recognition, and independently measured end-to-end latencies were 18.151 ms/image (95% CI: 18.10–18.20; P95: 19.74), 8.854 ms/crop (95% CI: 8.82–8.89; P95: 9.80), and 27.094 ms/image (95% CI: 27.03–27.16; P95: 28.96), respectively. The one-decimal values reported elsewhere are 18.2, 8.9, and 27.1 ms. Confidence intervals for sample-based detection metrics and mean latency were obtained by resampling the original test-image records, whereas Wilson intervals were used for exact-match proportions.

## 4. Results

### 4.1. Detection Performance Comparison

To evaluate the effectiveness of the detection model, the traditional method, Faster R-CNN–ResNet50–FPN [32], YOLOv5n [17], YOLOv8n [18], DBNet–ResNet50 [33], YOLO11n [16], and the proposed improved YOLO11 were compared. The results are presented in Table 4.

As shown in Table 4, the traditional method was affected by cluttered backgrounds and character interference. Faster R-CNN–ResNet50–FPN and DBNet-ResNet50 provided relatively strong localization performance but had higher trainable parameter counts and lower inference speeds than the lightweight YOLO detectors. Among the evaluated methods, the proposed detector obtained the highest observed mAP@0.5 and recall while maintaining 55 FPS. The confidence intervals overlap for some comparisons; therefore, the results are interpreted as observed performance differences rather than claims of statistical superiority.

Compared with the YOLO11n baseline, the proposed method improved mAP@0.5 and recall by 2.1 and 1.1 percentage points, respectively, while maintaining an inference speed of 55 FPS. The YOLO11n baseline contained 2.59 million trainable parameters, whereas the proposed detector contained 3.02 million trainable parameters. The increase of 434,256 parameters, corresponding to approximately 16.8%, resulted from the combined structural modifications, while cross-scale stem sharing in SED-Head and removal of the P5 detection output limited redundant parameter growth.

### 4.2. Recognition Performance Comparison

The original CRNN, ASTER, SVTR, MobileNetV3 + LSTM + CTC, and the proposed improved CRNN were compared on the recognition task [9,10,11,14,28,29]. The results are reported in Table 5.

As shown in Table 5, the original CRNN had the largest model size and the highest mean latency among the evaluated recognizers. ASTER and SVTR also had larger model files and higher mean latencies than the MobileNetV3-based configurations.

The proposed improved CRNN achieved an exact-match accuracy of 97.6%, a model size of 6.2 MB, and an inference time of 8.9 ms/crop.

### 4.3. Ablation Study

To examine the incremental effects of the cumulative model configurations, progressive ablation experiments were conducted for both the detection and recognition branches. At each cumulative stage, the newly listed component or component group was added to the preceding configuration, while the dataset division, input size, training schedule, and test environment were kept unchanged. The final detection-side stage evaluates SED-Head and DEConv as a combined configuration; their individual effects are not separated in the present ablation results.

#### 4.3.1. Detection-Side Ablation

All detection-side ablation experiments used the same dataset division, training parameters, input size, and test environment. YOLO11n was adopted as the baseline, followed cumulatively by C3k2-CSMA, HEFFPN, SBA, and the combined SED-Head and DEConv stage. The results are presented in Table 6.

The cumulative configurations produced progressively higher observed point estimates. After C3k2-CSMA was introduced, mAP@0.5 and recall reached 96.0% and 96.3%, respectively. The configuration including HEFFPN reached 96.6% for both metrics, and the configuration further including SBA reached 96.9% mAP@0.5 and 96.8% recall. These changes are consistent with the intended roles of multiscale representation, high-resolution fusion, and boundary-related feature refinement. Because the confidence intervals overlap, the incremental differences are not interpreted as statistically significant.

The final cumulative configuration incorporating SED-Head and DEConv achieved 97.3% mAP@0.5, 97.1% recall, and 55 FPS. Because SED-Head and DEConv were evaluated together in this stage, the present results do not isolate their individual contributions.

#### 4.3.2. Recognition-Side Ablation

All recognition-side cumulative configurations used the same training, validation, and test sets, input size, number of training epochs, and test environment. The MobileNetV3 + LSTM + CTC configuration replaces the original VGG-style feature-extraction backbone with MobileNetV3 while retaining LSTM-based sequence modeling and CTC decoding. The next cumulative configuration uses bidirectional LSTM sequence modeling, and the final configuration adds explicit L2 regularization. The results are reported in Table 7.

Introducing MobileNetV3 reduced the model size from 138.0 MB to 6.0 MB and the mean inference latency from 28.5 to 8.5 ms/crop, while the observed exact-match accuracy reached 97.2%. Adding Bi-LSTM increased the point estimate to 97.5%, and the final setting with L2 regularization obtained 97.6%. Because the confidence intervals overlap and the final L2 step corresponds to one additional correct sample, these results are interpreted as observed configuration differences rather than evidence of statistical superiority.

### 4.4. End-to-End System Performance

The performance of the complete two-stage system is summarized in Table 8.

As shown in Table 8, the complete system correctly recognized 1162 of 1200 test images, giving an end-to-end exact-match accuracy of 96.8% (95% CI: 95.7–97.7). Of the 1165 detections matched at IoU ≥ 0.50, 1137 produced correct complete sequences and 28 produced recognition errors. Among the remaining 35 samples, 25 predicted boxes had IoU below 0.50 but still preserved all 11 characters and produced correct sequences; the other 10 samples produced end-to-end errors. Thus, the fixed-IoU detection recall and end-to-end exact-match accuracy describe related but non-nested outcomes.

The detector and recognizer FP32 state_dict files occupied 12,184,736 bytes (12.2 MB) and 6,238,756 bytes (6.2 MB), respectively, giving a complete-system size of 18,423,492 bytes (18.4 MB). The complete pipeline contained approximately 4.58 M parameters, required approximately 10.25 GFLOPs for one image with one recognition crop, and reached 1.14 GiB peak allocated GPU memory during FP32 inference. Its independently measured mean end-to-end latency was 27.094 ms/image (95% CI: 27.03–27.16; P95: 28.96).

### 4.5. Complex-Scene Evaluation

To further evaluate performance under complex conditions, grouped evaluation was conducted using the overlapping test-set attributes listed in Table 2. Table 9 reports image-level 95% confidence intervals for the proposed method, YOLO11n, and DBNet-ResNet50. Because a test image may belong to more than one subset, the grouped results describe within-subset performance and are not treated as independent cross-group samples.

As shown in Table 9, the proposed method obtained the highest observed mAP@0.5 and recall point estimates across all evaluated subsets. For normal and clear samples, it achieved 98.7% mAP@0.5 and 98.5% recall. Under partial occlusion, the corresponding point estimates were 96.3% and 96.2%. Because several confidence intervals overlap, these comparisons are not presented as evidence of statistical superiority for every subset.

At the end-to-end level, exact-match accuracy was 98.5% [96.5, 99.4] for normal and clear samples, 95.9% [92.9, 97.6] for character degradation, and 95.4% [90.3, 97.9] for partial occlusion. The lower point estimates in the latter two subsets indicate that missing or degraded character evidence remains a practical limitation of the current two-stage method.

### 4.6. Sensitivity Analysis

Sensitivity was evaluated for the C3k2-CSMA branch-width ratio and the explicit L2 coefficient while keeping the remaining configuration fixed. The results for the C3k2-CSMA branch-width ratio are presented in Table 10.

Among the evaluated settings, ρ = 0.25 produced the highest observed mAP@0.5 and recall point estimates and retained a mean latency of 18.2 ms/image. It was therefore selected as the preferred accuracy–speed trade-off within the evaluated range; the overlapping intervals do not establish an absolute or statistically significant optimum.

The results for the explicit L2 regularization coefficient are presented in Table 11.

The coefficient λ = 1 × 10^−4^ produced the highest observed exact-match accuracy and was used for the final recognizer. The difference from λ = 0 corresponded to one additional correct sequence and should therefore be interpreted cautiously. The lower point estimate at λ = 1 × 10^−3^ indicates that a stronger constraint did not improve recognition under the evaluated setting.

### 4.7. Error and Boundary-Case Analysis

Table 12 summarizes four traceable records selected from the final test results. They include recognition errors following matched detections, a low-confidence candidate filtered before recognition, and a geometrically unmatched box that nevertheless retained the complete sequence.

The first two records show that exact-sequence errors can remain after a detection is counted as correct. In test_0648, the detector returned a candidate detection with confidence 0.22, which was removed by the operating threshold of 0.25 and therefore was not passed to the recognizer. Conversely, test_0974 shows that an IoU below 0.50 does not necessarily remove character information: the offset box retained all 11 characters and yielded the correct sequence.

## 5. Discussion

### 5.1. Mechanism Analysis of Performance Improvement

The detection ablation configurations are cumulative. The changes observed after C3k2-CSMA and HEFFPN are consistent with their intended roles in multiscale representation and high-resolution feature fusion, while the change after SBA is consistent with boundary refinement. The final stage jointly adds SED-Head and DEConv: SED-Head shares parameters across scales, whereas DEConv is reparameterized as a standard convolution for deployment. Because the two components were introduced together, their individual contributions cannot be separated by the current ablation.

On the recognition side, MobileNetV3 reduces model size and latency, Bi-LSTM adds bidirectional context, and L2 regularization defines the selected final configuration. These results are interpreted as observed differences within cumulative configurations. Where confidence intervals overlap, they are not treated as evidence of statistical superiority.

### 5.2. Efficiency and Deployment Analysis

The engineering objective was to balance detection accuracy with parameter count, computation, memory, storage, and measured speed. Relative to YOLO11n, the proposed detector increased trainable parameters from 2.59 M to 3.02 M (+16.8%) and FLOPs from 6.5 to 10.2 GFLOPs (+56.9%). Under the stated training conditions, peak allocated GPU memory increased from 3.74 to 5.86 GiB; during batch-size-1 FP32 inference, it increased from 0.82 to 1.11 GiB. Despite these costs, the measured throughput remained 55 FPS, compared with 54 FPS for YOLO11n. Shared processing and removal of the P5 prediction output constrained parameter duplication, but the high-resolution P2 path still produced a measurable computation and memory overhead.

The final recognizer contained 1.55 M parameters, required 0.0513 GFLOPs per crop, occupied 6.2 MB, and reached 0.07 GiB peak allocated memory with a mean latency of 8.9 ms/crop. For one 640 × 640 image containing one recognition region, the complete two-stage pipeline contained approximately 4.58 M parameters, required approximately 10.25 GFLOPs, occupied 18.4 MB, and reached 1.14 GiB peak allocated memory. Its independently measured end-to-end latency was 27.1 ms/image rather than a value calculated by adding the rounded stage-level measurements.

Trainable parameter count, FP32 model size, FLOPs, peak allocated GPU memory, and measured latency describe different aspects of complexity. FLOPs characterize theoretical operation count under the stated input, whereas allocated memory and latency depend on the software stack, hardware, batch size, and execution path. The current measurements therefore support comparison on the RTX 3060 platform but do not establish power consumption or latency on a target edge device.

### 5.3. Limitations and Future Work

First, the dataset consists of publicly accessible container-number images and additionally curated public scene images. Duplicate and obviously repeated samples were screened during data curation; however, visually similar samples originating from public image sources cannot be completely excluded. Independent external testing across different land-port yards, camera devices, and continuous acquisition periods has not yet been conducted. The reported results therefore characterize the evaluated dataset and acquisition conditions.

Second, the current speed, latency, and peak-memory measurements were obtained using an NVIDIA GeForce RTX 3060 12 GB GPU with a batch size of 1. They characterize relative efficiency under a unified experimental platform but cannot be directly interpreted as on-site performance on an edge device. Long-term operational stability, power consumption, memory behavior on target edge hardware, and performance under different inference backends have not yet been evaluated. A dedicated comparison of convergence behavior, gradient statistics, and hyperparameter-search cost was not conducted; the reported sensitivity analysis was limited to ρ and λ under the stated training protocol.

Third, end-to-end recognition accuracy was calculated using the strict criterion that the final decoded sequence must match all 11 characters. Although this metric reflects the proportion of valid system-level outputs, supplementary metrics such as character-level accuracy, edit distance, and confidence calibration have not yet been reported. The representative records in Table 12 and the lower grouped accuracies for partial occlusion and character degradation show that detection geometry, threshold filtering, and degraded character evidence can produce different failure paths. Strong specular reflection was not separately quantified and should be included as a dedicated condition in future testing.

Future work will focus on continuous data collection in real land-port environments, cross-domain external validation, and long-term deployment on edge devices. Multi-frame information fusion, image enhancement, and joint optimization of the detection and recognition branches will be investigated as future research directions.

## 6. Conclusions

This study proposed a two-stage detection–cropping–recognition method for container-number identification in complex land-port yard scenes. The improved YOLO11 detector achieved 97.3% mAP@0.5, 97.1% recall, and 55 FPS, with 3.02 M trainable parameters, 10.2 GFLOPs, and an FP32 model size of 12.2 MB. The lightweight CRNN recognizer achieved 97.6% exact-match accuracy, a model size of 6.2 MB, and a mean latency of 8.9 ms/crop. The complete system obtained 96.8% end-to-end exact-match accuracy, required approximately 10.25 GFLOPs for one image with one recognition crop, occupied 18.4 MB, and achieved a mean end-to-end latency of 27.1 ms/image.

However, the current evaluation was based on publicly accessible images and an NVIDIA GeForce RTX 3060 GPU, without independent cross-port testing or edge-device validation. Future work will therefore focus on cross-domain external evaluation, edge-device testing, and multi-frame information fusion.

## Figures and Tables

**Figure 1:**
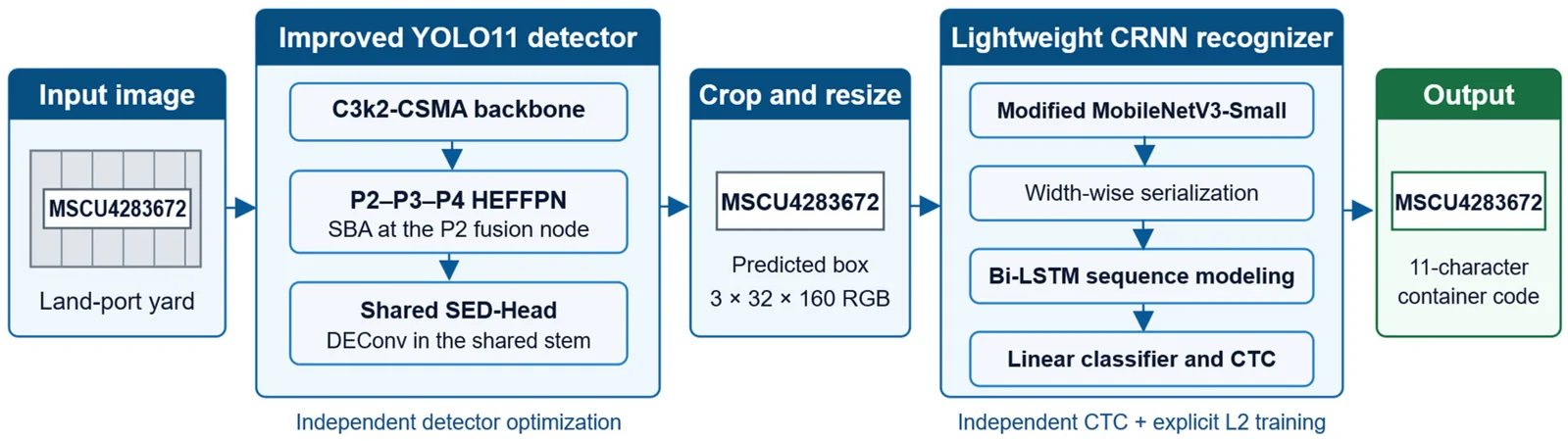
Overall framework of the proposed container-number detection and recognition method.

**Figure 2:**
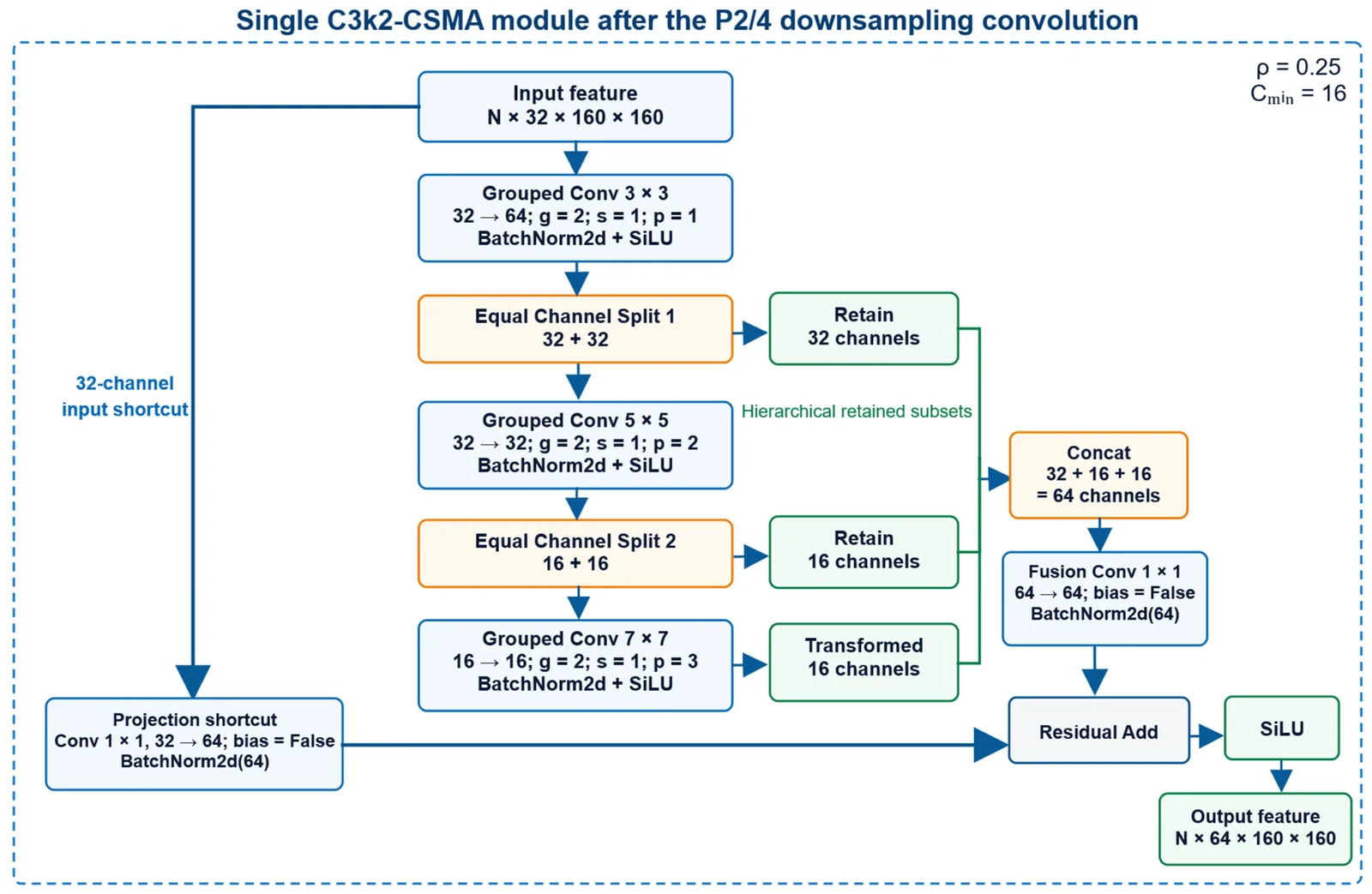
Fixed implementation of the single C3k2-CSMA module inserted after the P2/4 downsampling convolution. Successive equal channel splits route 32-, 16-, and 16-channel features to concatenation, followed by 1 × 1 fusion and a projected residual shortcut.

**Figure 3:**
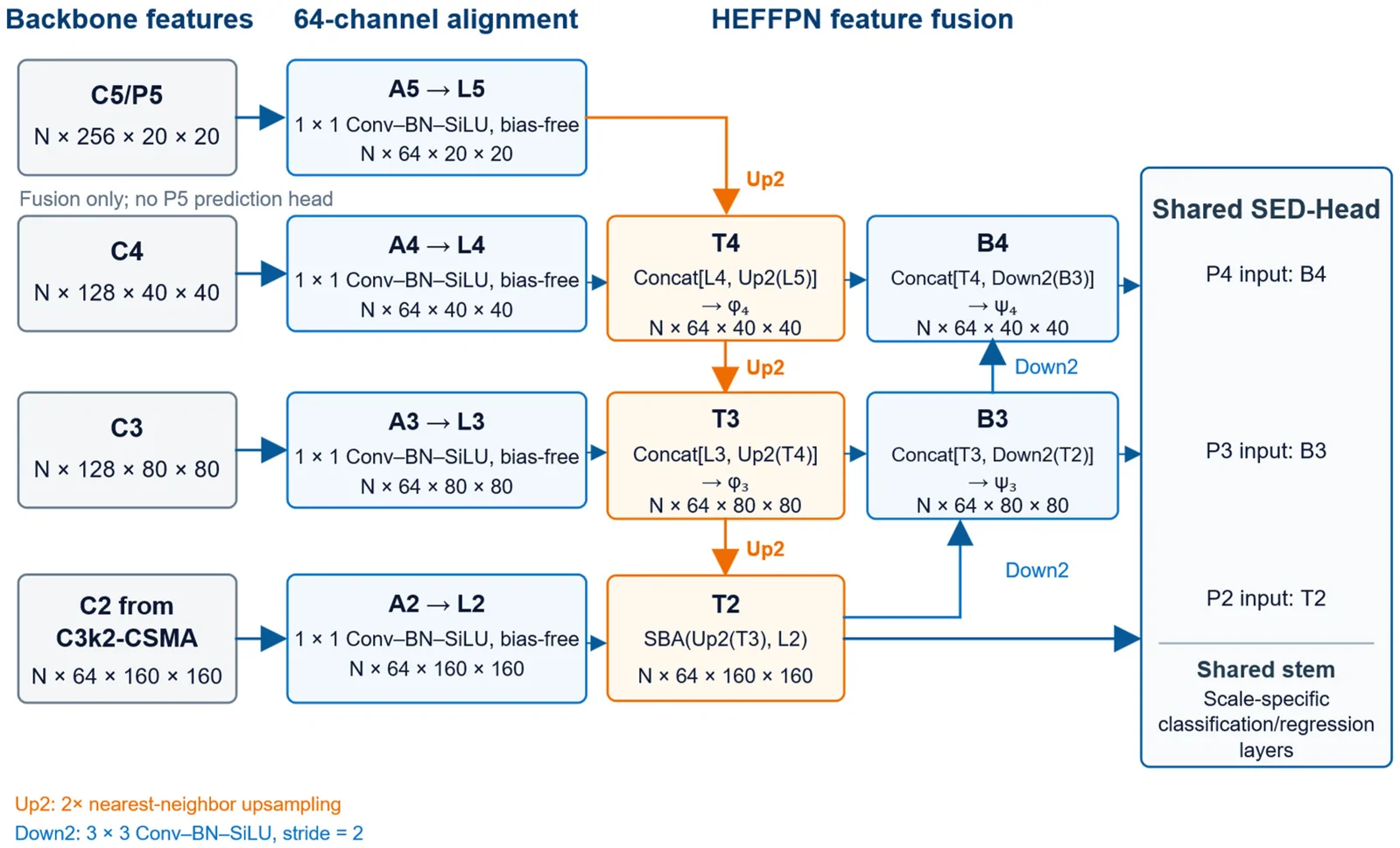
Fixed HEFFPN topology and detection-scale reconfiguration. The C5/P5 backbone feature is retained as the top-down semantic input without a P5 detection head; the top-down path runs from P5 to P2, and the bottom-up path runs from P2 to P4. The shared SED-Head receives T2, B3, and B4 at the P2, P3, and P4 scales, respectively.

**Figure 4:**
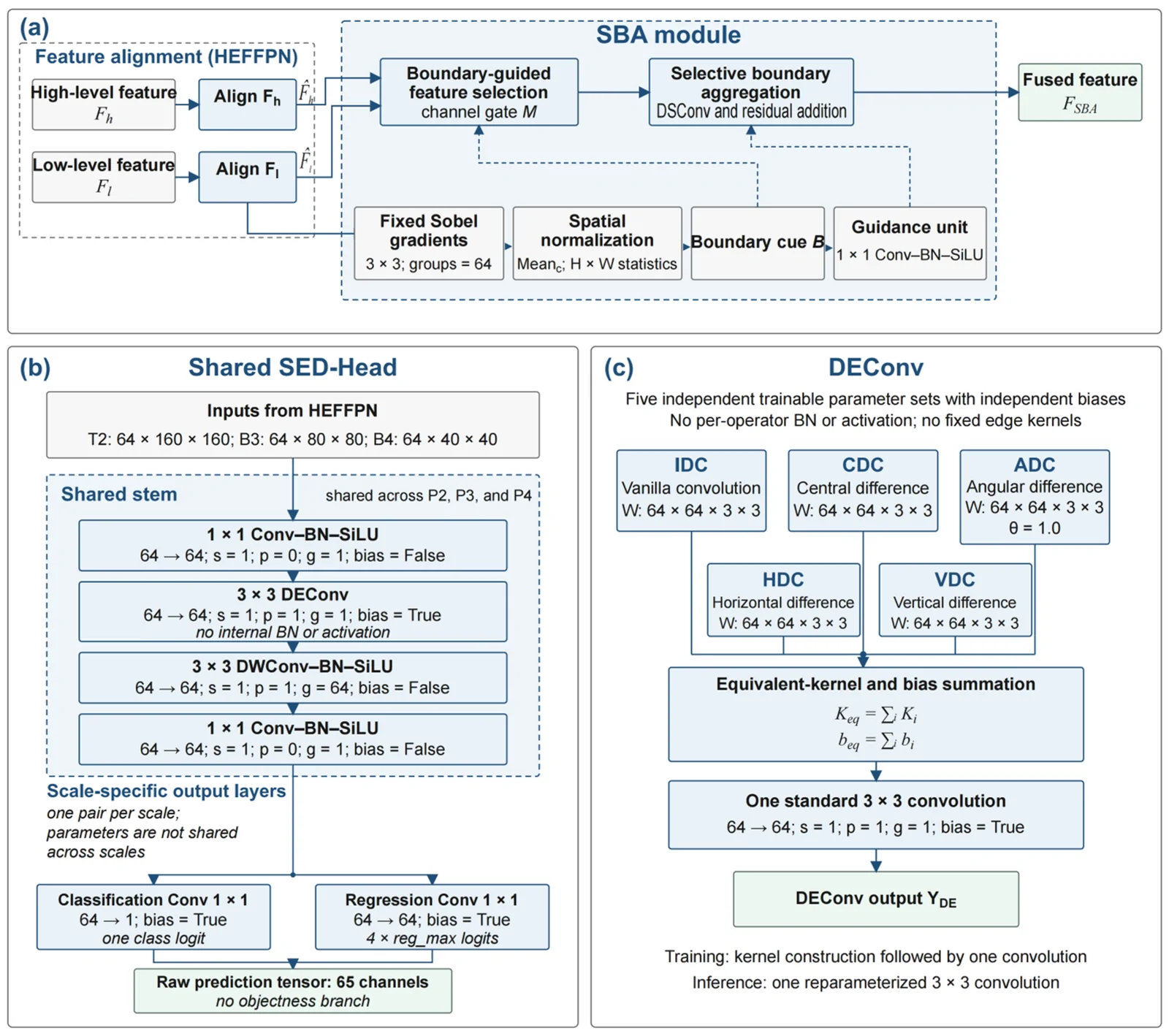
Architectures of the proposed detection enhancement components: (**a**) SBA at the P2 HEFFPN fusion node, including internal Sobel boundary-cue generation; (**b**) SED-Head with one stem shared across P2, P3, and P4 and scale-specific classification and regression output layers; (**c**) DEConv with VC, CDC, ADC, HDC, and VDC parameterizations, equivalent-kernel and bias summation, and a single reparameterized 3 × 3 convolution.

**Figure 5:**
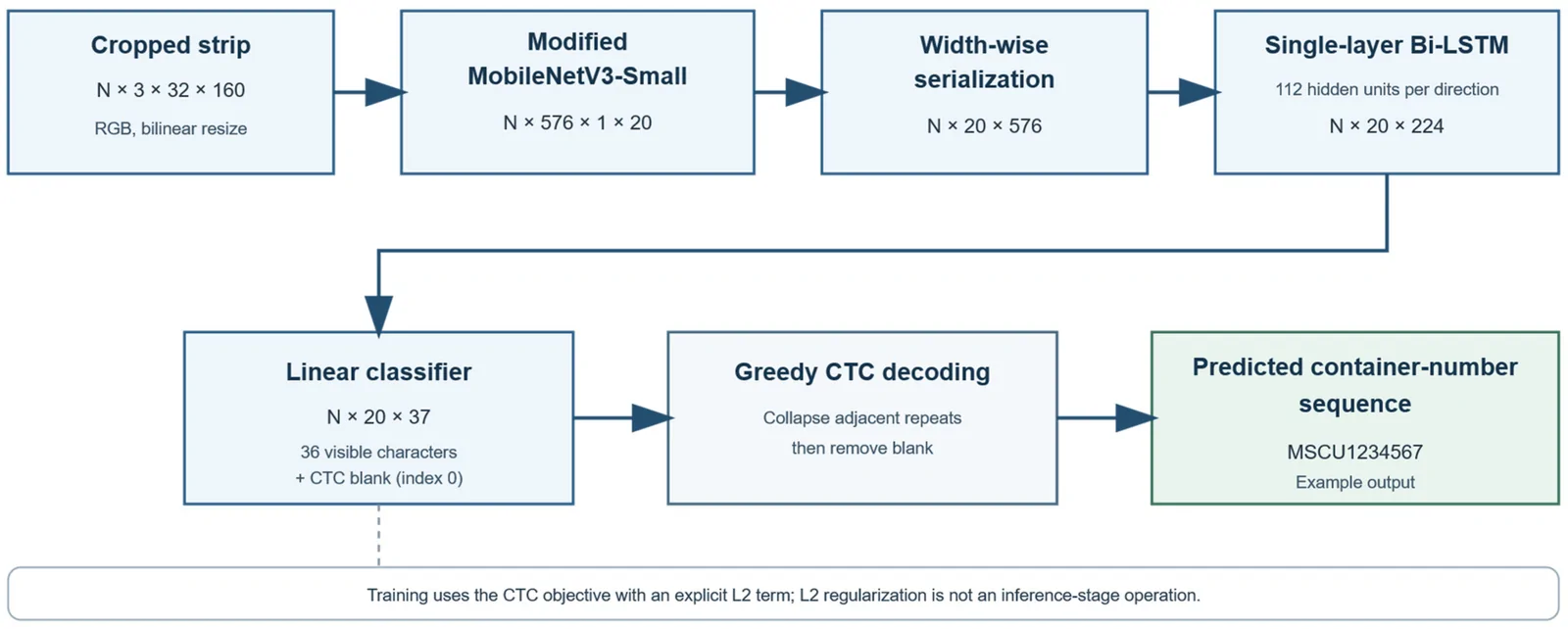
Architecture of the lightweight CRNN recognition model.

**Figure 6:**
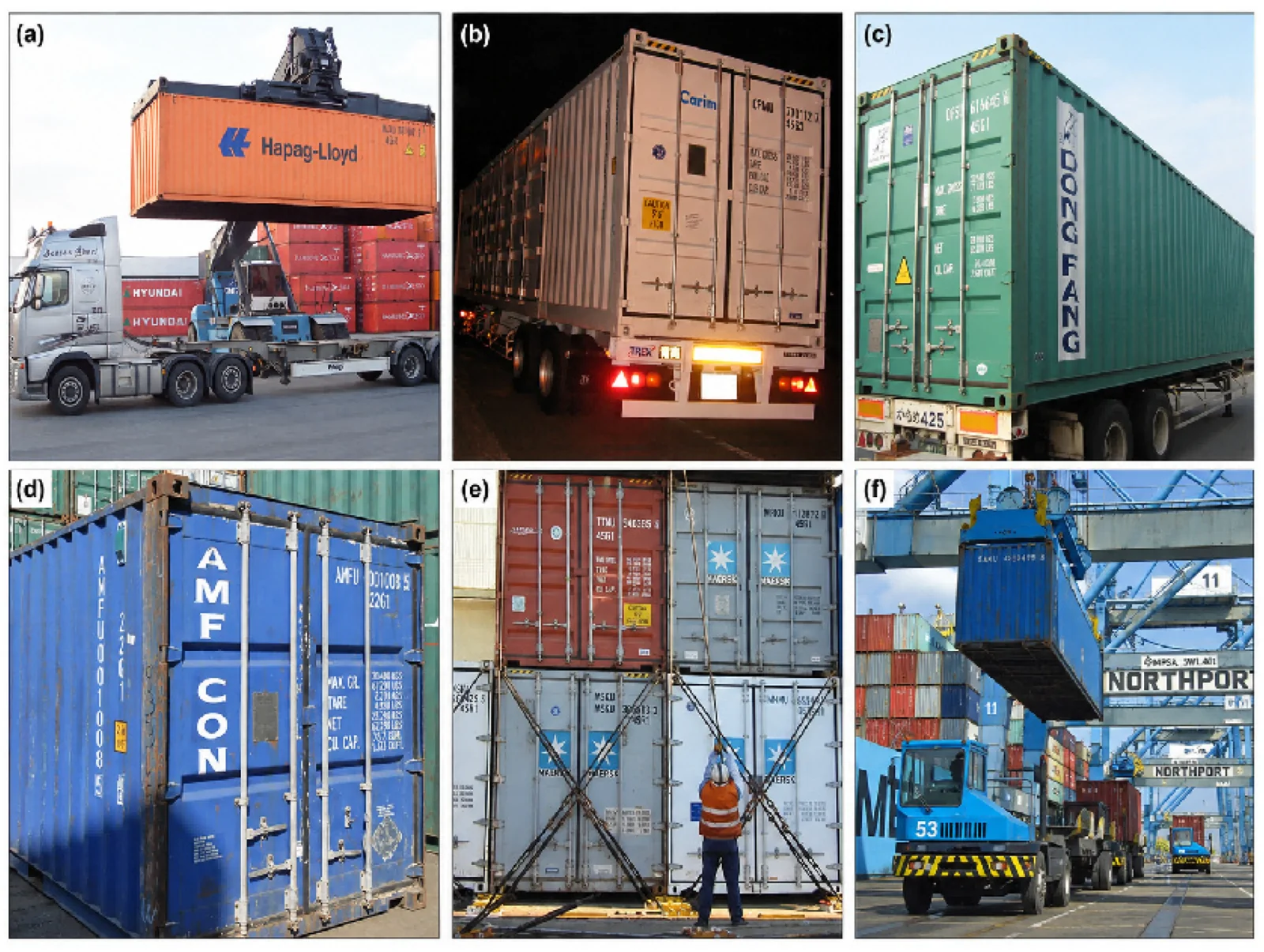
Examples of container-number detection in complex scenes: (**a**) distant small-scale target; (**b**) illumination variation; (**c**) viewpoint variation; (**d**) character degradation; (**e**) partial occlusion; and (**f**) background-character or structural interference.

**Table 1:** Dataset division.

Data Subset	Number of Images	Proportion (%)	Purpose	Random Augmentation
Training set	1920	53.3	Model training	Yes
Validation set	480	13.3	Model selection and hyperparameter adjustment	No
Test set	1200	33.3	Final performance evaluation	No
Total	3600	100.0	—	—

Percentages were independently rounded to one decimal place and may not sum to exactly 100.0%.

**Table 2:** Multi-label statistics of complex-scene attributes.

Scene Attribute	Training Set	Validation Set	Test Set	Total Attribute Samples
Normal and clear	520	130	330	980
Distant small-scale target	460	115	285	860
Illumination variation	300	75	185	560
Viewpoint variation	360	90	230	680
Character degradation	470	115	290	875
Partial occlusion	210	50	130	390
Background-character or structural interference	610	150	380	1140

**Table 3:** Experimental environment and principal training parameters.

Item	Setting
Operating system	Windows 10 64-bit
CPU	Intel Core i7-12700
GPU	NVIDIA GeForce RTX 3060 12 GB
Memory	32 GB
Deep learning framework	PyTorch 2.1.1
Ultralytics version	8.3.0
TorchVision version	0.16.1
CUDA/cuDNN	CUDA 11.8/cuDNN 8.9
Programming language	Python 3.10
Detection input size	640 × 640
Recognition input size	32 × 160
Recognition preprocessing	RGB; direct bilinear resize to 32 × 160; pixel scaling to [0, 1]; ImageNet normalization
Recognition backbone	Modified MobileNetV3-Small; ImageNet-1K initialization; all feature layers fine-tuned
Bi-LSTM	One layer; 112 hidden units per direction; bidirectional; dropout = 0
Character classification and decoding	37 classes including CTC blank; blank index = 0; greedy CTC decoding
Recognition regularization	Explicit L2 coefficient = 1 × 10^−4^; Adam weight_decay = 0
Detection training	100 epochs; batch size 8; initial learning rate 0.01; SGD; seed = 0
Recognition training	100 epochs; batch size 64; initial learning rate 0.001; Adam; seed = 0
Runtime measurement	NVIDIA GeForce RTX 3060 12 GB; batch size 1; FP32; 50 warm-up iterations; CUDA synchronization; disk reading, result saving, and visualization excluded

**Table 4:** Comparison of detection models.

Method	Type	mAP@0.5 (%, 95% CI)	Recall (%, 95% CI)	FPS (95% CI)	Parameters (M)
Connected components + template matching	Traditional method	86.4 [84.2, 88.5]	85.5 [83.4, 87.4]	18.0 [17.9, 18.1]	—
Faster R-CNN–ResNet50–FPN	Two-stage detector	91.8 [90.1, 93.4]	92.4 [90.8, 93.8]	12.0 [11.9, 12.1]	41.30
YOLOv5n	Lightweight YOLO detector	93.6 [92.2, 94.9]	94.1 [92.6, 95.3]	61.0 [60.6, 61.4]	1.77
YOLOv8n	Lightweight YOLO detector	94.8 [93.5, 95.9]	95.0 [93.6, 96.1]	57.0 [56.7, 57.4]	3.14
DBNet-ResNet50	Scene-text detector	95.6 [94.4, 96.6]	95.3 [94.0, 96.4]	26.0 [25.8, 26.2]	24.72
YOLO11n	Baseline model	95.2 [93.9, 96.3]	96.0 [94.7, 97.0]	54.0 [53.6, 54.3]	2.59
Proposed improved YOLO11	Proposed method	97.3 [96.4, 98.1]	97.1 [96.0, 97.9]	55.0 [54.9, 55.2]	3.02

**Table 5:** Comparison of recognition models.

Method	Type	Exact-Match Accuracy (%, 95% CI)	Model Size (MB)	Mean Latency (ms/Crop, 95% CI)
Original CRNN	CTC baseline model	96.8 [95.7, 97.7]	138.0	28.5 [28.38, 28.62]
ASTER	Attention-based recognizer	97.1 [96.0, 97.9]	45.8	18.6 [18.52, 18.68]
SVTR	Transformer-based recognizer	97.4 [96.4, 98.2]	24.6	12.3 [12.25, 12.35]
MobileNetV3 + LSTM + CTC	Lightweight baseline model	97.2 [96.1, 98.0]	6.0	8.5 [8.47, 8.53]
Proposed improved CRNN	Proposed method	97.6 [96.6, 98.3]	6.2	8.9 [8.82, 8.89]

**Table 6:** Ablation results for the detection modules.

Model Configuration	mAP@0.5 (%, 95% CI)	Recall (%, 95% CI)	FPS (95% CI)
YOLO11n	95.2 [93.9, 96.3]	96.0 [94.7, 97.0]	54.0 [53.6, 54.3]
+C3k2-CSMA	96.0 [94.9, 97.0]	96.3 [95.1, 97.3]	53.0 [52.7, 53.3]
+HEFFPN	96.6 [95.5, 97.5]	96.6 [95.4, 97.5]	52.0 [51.7, 52.3]
+SBA	96.9 [95.9, 97.7]	96.8 [95.7, 97.7]	51.0 [50.7, 51.3]
+SED-Head and DEConv (combined stage)	97.3 [96.4, 98.1]	97.1 [96.0, 97.9]	55.0 [54.9, 55.2]

**Table 7:** Ablation results for the recognition modules.

Model Configuration	Exact-Match Accuracy (%, 95% CI)	Model Size (MB)	Mean Latency (ms/Crop, 95% CI)
Original CRNN	96.8 [95.7, 97.7]	138.0	28.5 [28.38, 28.62]
MobileNetV3 + LSTM + CTC	97.2 [96.1, 98.0]	6.0	8.5 [8.47, 8.53]
MobileNetV3 + Bi-LSTM + CTC	97.5 [96.5, 98.2]	6.2	8.8 [8.77, 8.83]
MobileNetV3 + Bi-LSTM + CTC + L2 regularization	97.6 [96.6, 98.3]	6.2	8.9 [8.82, 8.89]

**Table 8:** End-to-end system performance and complexity summary.

Metric	Value
End-to-end exact-match accuracy (%, 95% CI)	96.8 [95.7, 97.7]
Detector/recognizer/total parameters (M)	3.02/1.55/4.58
Detector/recognizer/total GFLOPs	10.2/0.0513/approximately 10.25
Detector/recognizer/total model size (MB)	12.2/6.2/18.4
YOLO11n/proposed-detector training peak allocated memory (GiB)	3.74/5.86
YOLO11n/proposed-detector inference peak allocated memory (GiB)	0.82/1.11
Recognizer/complete-pipeline inference peak allocated memory (GiB)	0.07/1.14
Detection latency (ms/image; 95% CI; P95)	18.2 [18.10, 18.20]; 19.74
Recognition latency (ms/crop; 95% CI; P95)	8.9 [8.82, 8.89]; 9.80
End-to-end latency (ms/image; 95% CI; P95)	27.1 [27.03, 27.16]; 28.96

**Table 9:** Performance comparison under different complex-scene attributes.

Test Subset	Model	mAP@0.5 (%, 95% CI)	Recall (%, 95% CI)	End-to-End Exact-Match Accuracy (%, 95% CI)
Normal and clear (*n* = 330)	YOLO11n	97.4 [95.4, 98.6]	97.6 [95.3, 98.8]	—
	DBNet-ResNet50	97.8 [96.0, 98.9]	97.3 [94.9, 98.6]	—
	Proposed	98.7 [97.2, 99.4]	98.5 [96.5, 99.4]	98.5 [96.5, 99.4]
Viewpoint variation (*n* = 230)	YOLO11n	95.1 [91.9, 97.3]	95.2 [91.6, 97.3]	—
	DBNet-ResNet50	95.8 [92.9, 97.7]	95.2 [91.6, 97.3]	—
	Proposed	97.4 [95.0, 98.7]	97.0 [93.9, 98.5]	96.5 [93.3, 98.2]
Background/structural interference (*n* = 380)	YOLO11n	94.5 [91.9, 96.4]	94.7 [92.0, 96.6]	—
	DBNet-ResNet50	95.7 [93.4, 97.3]	95.0 [92.3, 96.8]	—
	Proposed	97.2 [95.3, 98.3]	96.8 [94.6, 98.2]	96.6 [94.2, 98.0]
Illumination variation (*n* = 185)	YOLO11n	94.6 [90.6, 97.0]	95.1 [91.0, 97.4]	—
	DBNet-ResNet50	95.2 [91.4, 97.5]	94.6 [90.3, 97.0]	—
	Proposed	97.1 [94.0, 98.6]	96.8 [93.1, 98.5]	96.8 [93.1, 98.5]
Distant small-scale target (*n* = 285)	YOLO11n	93.8 [90.5, 96.1]	94.0 [90.7, 96.2]	—
	DBNet-ResNet50	94.6 [91.5, 96.7]	94.0 [90.7, 96.2]	—
	Proposed	96.8 [94.2, 98.2]	96.5 [93.7, 98.1]	96.1 [93.2, 97.8]
Character degradation (*n* = 290)	YOLO11n	94.2 [91.0, 96.4]	94.5 [91.2, 96.6]	—
	DBNet-ResNet50	95.0 [92.0, 97.0]	94.1 [90.8, 96.3]	—
	Proposed	96.9 [94.4, 98.3]	96.6 [93.8, 98.1]	95.9 [92.9, 97.6]
Partial occlusion (*n* = 130)	YOLO11n	93.0 [87.4, 96.4]	93.1 [87.4, 96.3]	—
	DBNet-ResNet50	93.8 [88.4, 97.0]	93.1 [87.4, 96.3]	—
	Proposed	96.3 [91.9, 98.3]	96.2 [91.3, 98.3]	95.4 [90.3, 97.9]

**Table 10:** Sensitivity to the C3k2-CSMA branch-width ratio.

Branch-Width Ratio ρ	mAP@0.5 (%, 95% CI)	Recall (%, 95% CI)	Latency (ms/Image, 95% CI)
0.125	97.0 [96.0, 97.8]	96.8 [95.7, 97.7]	17.9 [17.8, 18.0]
0.25	97.3 [96.4, 98.1]	97.1 [96.0, 97.9]	18.2 [18.1, 18.2]
0.50	97.1 [96.2, 97.9]	96.9 [95.8, 97.8]	19.2 [19.1, 19.4]

**Table 11:** Sensitivity to the explicit L2 regularization coefficient.

L2 Coefficient	Correct Sequences	Exact-Match Accuracy (%, 95% CI)
0	1170/1200	97.5 [96.5, 98.2]
1 × 10^−5^	1170/1200	97.5 [96.5, 98.2]
1 × 10^−4^	1171/1200	97.6 [96.6, 98.3]
1 × 10^−3^	1166/1200	97.2 [96.1, 98.0]

**Table 12:** Selected failure and boundary-case records from the final test set.

Sample ID	Scene/Outcome	Ground Truth	Confidence/IoU	Recognition Output	Observed Result
test_0117	Character degradation	MSCU1234566	0.94/0.82	MSCU1234586	Matched detection; 6/8 confusion
test_0236	Partial occlusion	TGHU7654320	0.89/0.71	TGHU765432	Matched detection; final character omitted
test_0648	Low-confidence candidate	CMAU2048179	0.22/0.38	Not evaluated	Filtered at confidence 0.25
test_0974	Boundary localization offset	OOLU9182731	0.76/0.44	OOLU9182731	IoU below 0.50; sequence correct

## Data Availability

The images used in this study were obtained from publicly accessible third-party sources. Because the authors do not hold redistribution rights for all source images, neither the original images nor the compiled image dataset is publicly available or provided by the authors. The processed annotations, dataset-split indices, and experimental records obtained during model evaluation are available from the corresponding author upon reasonable request.
